# Research progress on the mechanism of cholesterol-25-hydroxylase in intestinal immunity

**DOI:** 10.3389/fimmu.2023.1241262

**Published:** 2023-08-31

**Authors:** Guoqiang Zhong, Chengcheng He, Shanping Wang, Chuangzhen Lin, Mingsong Li

**Affiliations:** Inflammatory Bowel Diseases Research Center, Department of Gastroenterology, The Third Affiliated Hospital of Guangzhou Medical University, Guangzhou, Guangdong, China

**Keywords:** cholesterol-25-hydroxylase, 25-hydroxycholesterol, inflammatory bowel disease, intestinal immunity, mechanism

## Abstract

Inflammatory bowel disease (IBD), a general term encompassing Crohn’s disease (CD) and ulcerative colitis (UC), and other conditions, is a chronic and relapsing autoimmune disease that can occur in any part of the digestive tract. While the cause of IBD remains unclear, it is acknowledged that the disease has much to do with the dysregulation of intestinal immunity. In the intestinal immune regulatory system, Cholesterol-25-hydroxylase (CH25H) plays an important role in regulating the function of immune cells and lipid metabolism through catalyzing the oxidation of cholesterol into 25-hydroxycholesterol (25-HC). Specifically, CH25H focuses its mechanism of regulating the inflammatory response, signal transduction and cell migration on various types of immune cells by binding to relevant receptors, and the mechanism of regulating lipid metabolism and immune cell function via the transcription factor Sterol Regulator-Binding Protein. Based on this foundation, this article will review the function of CH25H in intestinal immunity, aiming to provide evidence for supporting the discovery of early diagnostic and treatment targets for IBD.

## 1 Introduction

Inflammatory bowel disease (IBD) including Crohn’s disease (CD) and ulcerative colitis (UC) is a chronic and relapsing inflammatory digestive disease. As the incidence of IBD has rapidly increased, an increasing number of experts and scholars are investigating the etiology of the disease. Although IBD is founded to be closely related to external factors such as people’s dietary habits and living environment (1), its internal factors, namely its specific cause and exact mechanism, remain to be explored. It is acknowledged that IBD is primarily associated with environmental factors, genetic predisposition, the dysbiosis of intestinal microbiota, and immune dysfunction. CH25H has been demonstrated to have the ability to regulate intestinal immunity and is associated with IBD (2). Previous studies have found that cholesterol-25-hydroxylase (CH25H) can catalyze the production of 25-hydroxycholesterol (25-HC) from cholesterol (3). Sterol regulatory-element binding proteins (SREBPs) is also conducive to transcription in the intestinal immune regulatory system. SREBPs are a family of transcriptive factors that regulate the expression of genes involved in lipogenic processes. They also get involved in numerous cellular processes and pathologies such as reactive oxygen species generation, endoplasmic reticulum stress, apoptosis, and autophagy (4). 25-HC can inactivate SREBPs to inhibit lipid synthesis or exert anti-inflammatory effects. In addition to its role in cholesterol metabolism, CH25H and its downstream products can bind to relevant receptors and participate in regulating immune system, including the direct regulation of inflammatory programming, the development of immune responses, and the signaling transduction. Previous studies have highlighted the significance of CH25H in immunology, arguing that CH25H plays a protective role against viral and bacterial infections (5). However, further investigation is needed to examine the function of CH25H in intestinal immunity, particularly in relation to IBD. Based on this foundation, this article is intended to provide an overview of the research progress of CH25H in intestinal immunity.

## 2 Biological characteristics of CH25H

**Figure 1:**
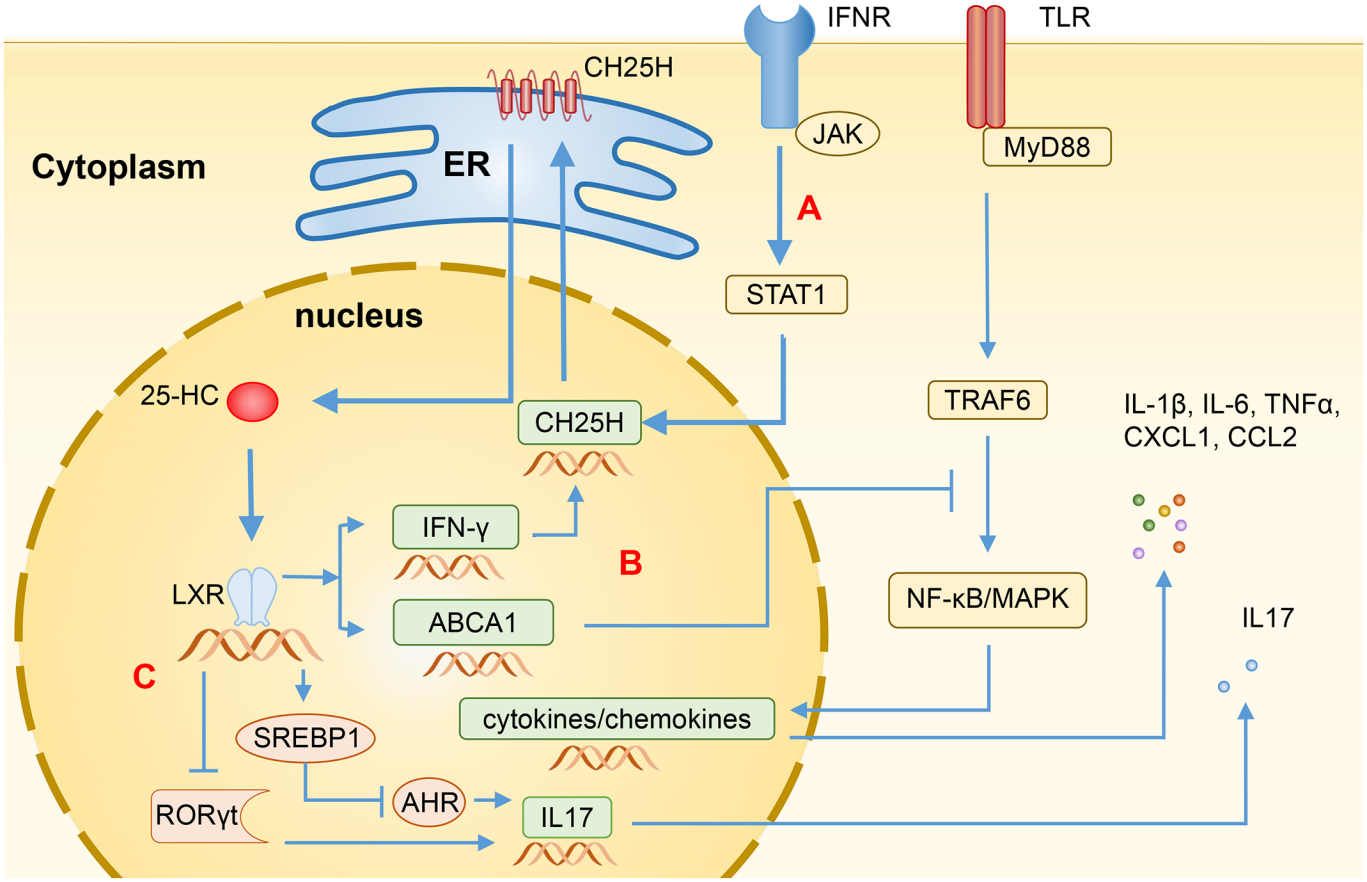
Regulation of immune function by 25-HC through LXR. **(A)** The transcription levels of CH25H are induced by Type I IFN through the STAT1-dependent signaling pathway, resulting in the production of 25-HC via cholesterol oxidation. **(B)** Binding LXR, 25-HC can induce the expression of ABCA1, disrupting the recruitment of MyD88 and TRAF6, and subsequently blocking the TLR-induced activation of NF-κB and MAPK, thereby suppressing the production of various inflammatory mediators such as IL-1β, IL-6, IL-17, TNFα, CXCL1, and CCL2. Additionally, 25-HC can also induce the expression of IFN-γ, thereby enhancing the CH25H expression through feedback regulation. **(C)** In CD4+ T cells, 25-HC activates SREBP1 or inhibits RORγt through LXR, leading to the inhibition of IL-17 expression and Th17 cell differentiation. 25-HC, 25-hydroxycholesterol; CH25H, Cholesterol-25-hydroxylase; LXR, Liver X receptor; IFN, Interferon; ABCA1, ATP-binding cassette transporters A1; MyD88, myeloid differentiation primary response protein 88; TRAF6, TNF Receptor Associated Factor 6; TLR, Toll-like receptor; NF-κB, Nuclear factor-κB, MAPK, Mitogen-activated protein kinase; TNFα, Tumor necrosis factor α; CXCL1, C-X-C motif chemokine ligand 1; CCL2, C-C motif chemokine ligand 2; SREBP1, Sterol regulatory-element binding protein 1; RORγt, Retinoic acid receptor-related orphan receptor γt; AHR, Aryl hydrocarbon receptor.

CH25H has some biological characteristics, including CH25H gene, CH25H protein, and 25-HC. Specifically, CH25H gene is a member of interferon-stimulating genes (ISGs), which has crucial functions in inflammation, innate immunity, and adaptive immune responses via interferon (IFN) signaling. CH25H protein belongs to the redox enzyme family and is a 31.6-kDa endoplasmic reticulum (ER)-associated hydroxylase that can catalyze cholesterol to produce 25-HC (6). 25-HC is an endogenous oxysterol produced by the oxidation of CH25H. Its function is to regulate the activity of SREBP, thereby inhibiting the biosynthesis of cholesterol and reducing its accumulation. Furthermore, 25-HC can also regulate the activity of nuclear receptors, which enables the regulation of immune response processes and lipid metabolism (3, 7). Regarding the regulation of CH25H expression, Diczfalusy et al. (8) have found that lipopolysaccharide (LPS) can induce an increase in the level of CH25H in macrophages of both mice and human volunteers. In a similar vein, Park et al. (9) conducted a further investigation, discovering that the expression of CH25H is mainly up-regulated by type I IFN via JAK/STAT1 signaling pathway. Through our observations, we have noted that the rapid induction of CH25H subsequently leads to the production of 25-HC, indicating that CH25H plays a significant role in lipid metabolism, gene expression, and immune activation (**Figure 1A**). Since previous studies have shown that CH25H plays a vital role in antiviral infections and various immune system-related diseases (3, 7), these regulatory mechanisms mentioned above have the possibility to be involved in the pathogenesis of IBD.

CH25H is widely expressed in various immune cells in physiological conditions, including macrophages, dendritic cells (DCs), neutrophils, and many others (10), whereas 25-HC is mainly produced in macrophages (11, 12) and regulates the functions and phenotypes of various immune cells, including macrophages, B cells, T cells, DCs, and neutrophils through its receptors (13). Notably, 25-HC has both pro-inflammatory and anti-inflammatory effects, and these differences may depend on its concentration and microenvironment, thereby exerting different functions in regulating gene expression, cellular proliferation, differentiation, and apoptosis (7).

## 3 Mechanism of CH25H regulation on intestinal immunity

The innate and adaptive immune responses in intestinal immune system are typically tightly regulated. Imbalances in the immune system will result in the development and progression of IBD (14). The immunological dysregulation present in IBD is characterized by epithelial damage, the expansion of inflammation facilitated by intestinal microbiota, and the infiltration of various types of cells into lamina propria, including T cells, B cells, macrophages, DCs, and neutrophils. These activated cells within the lamina propria produce proinflammatory cytokines such as tumor necrosis factor (TNF), Interleukin-1β (IL-1β), IFN-γ, and the IL-6 family of cytokines, leading to a high level of inflammation in the local tissue (15). CH25H can potentially contribute to the pathogenesis of IBD as it is widely expressed in various immune cells (10, 13). Intestinal dysbiosis and IBD often have a mutually causal relationship. The dysbiosis observed in individuals with IBD is associated with the increased production of LPS, and damage to the gut epithelial barrier allows LPS produced by the microbiota to enter the bloodstream, resulting in metabolic endotoxemia (16). Upon LPS stimulation, the TLR4/TRIF dependent pathway upregulates the expression of CH25H (8). The CH25H metabolite can act on multiple membrane receptors and nuclear receptors, exerting regulatory functions. Among these receptors, the liver X receptor (LXR), G protein-coupled receptor 183 (GPR183), and retinoic acid receptor-related orphan receptor (ROR) are pivotal in immune regulation.

### 3.1 LXR

LXRs, as the nuclear receptor of ligand-activated transcription factors, is capable of participating in various metabolic processes, including lipid, cholesterol, and carbohydrate metabolism. There are two LXR isoforms: LXRα and LXRβ (7, 17). LXRα may primarily play a role in the innate immune response via macrophages and dendritic cells, whereas LXRβ is likely responsible for the immune response in colonic epithelial cells and the adaptive immune response through CD3/CD4 lymphocytes (17). The activation of LXRs in a mouse colitis model can suppress the expression of inflammatory factors such as IL-1β, IL-6, IL-17A, TNFα, and chemokines such as CXCL1/KC, CXCL2/MIP2, CXCL8/IL-8, CCL2/MCP1 and CXCL10 in the colon tissue (17). In addition, LXRs can aid in the polarization of M2 macrophages and enhance the phagocytosis of macrophages and DCs, thereby increasing the clearance of senescent neutrophils and maintaining the neutrophil homeostasis (18, 19).

Being 25-HC an LXR ligand, it can induce the expression of cholesterol efflux transporters, such as ATP-binding cassette transporters A1 (ABCA1) and ATP-binding cassette transporters G1 (ABCG1). 25-HC can also upregulate the expression of Apolipoprotein E (ApoE) and Cytochrome P450 family 7 subfamily A member 1 (CYP7A1), which are important for cholesterol clearance (20). Additionally, 25-HC can induce the expression of IFN-γ in an LXR-dependent mechanism. The activated IFN-γ can subsequently enhance the expression of CH25H, forming a positive feedback loop (21). Ito et al. (22) have also found that the activation of LXRs can induce the expression of ABCA1, which could impede the recruitment of myeloid differentiation primary response protein 88 (MyD88) and TNF Receptor Associated Factor 6 (TRAF6), and subsequently block the Toll-like receptor (TLR)-induced activation of nuclear factor-κB (NF-κB) and mitogen-activated protein kinase (MAPK) signaling pathways. Consequently, this leads to the inhibition of the production of several inflammatory mediators, such as IL-1β, IL-6, IL-17, TNFα, CXCL1, and CCL2 (**Figure 1B**).

LXRs are associated with the innate immunity against pathogens. Research has shown that LXRs can protect macrophages from pathogen-induced cell death, thereby reducing pathogen infection (23, 24). There are two types of macrophages involving in host defense and tissue homoeostasis: M1 and M2 macrophages. M2 macrophages possess an anti-inflammatory property, contributing to maintaining tissue homeostasis. M2 macrophages can be polarized due to the activation of LXR by 25-HC. This polarization is generated due to the presence of intracellular amino-acid sufficiency signal and the extrinsic IL-4 signal (19). On the other hand, under the stimulation of pathogenic microorganisms, IFN-γ or TLR ligands can activate macrophages to become M1 macrophages, which play a role in initiating and sustaining inflammation (19). However, both IFN-γ or TLR ligands can also lead to the production of 25-HC, which can promote the polarization of M2 macrophages. It has been reported that the activation of TLR4 by LPS can increase the level of 25-HC, thereby regulating the LXR-dependent lipid metabolism and immune response, and inhibiting the expression of inflammatory mediators in macrophages such as COX-2, IL-6, IL-1β, and G-CSF (25).

LXRs also affect the function of neutrophils. Smoak et al. (26) have shown that the activation of LXRs can inhibit neutrophil motility, thus impairing pulmonary antibacterial host defense, and worsening the survival of mice. Korf et al. (27) found that mice lacking LXRs showed a deficiency in the early neutrophilic airway response to Mycobacterium tuberculosis infection, an impaired Th1/Th17 function, and a higher susceptibility to disseminated systemic infection. Reboldi et al. (28) discovered that the recruitment of neutrophils to the peritoneum relies on IL-1β, whereas 25-HC functions in suppressing IL-1β-mediated peritoneal inflammation by acting the downstream of type I IFN. The findings indicate that the signaling pathway of LXRs, which is activated by 25-HC, plays multiple roles in the regulation of innate immunity.

Anti-aging gene Sirtuin 1 (SIRT1) is associated with autoimmune diseases and irreversible programmed cell death in various cells and tissues (29). SIRT1 is regulated by environmental factors, diet, stress, lifestyle factors and bacterial LPS (30, 31). SIRT1 has to do with LXRs, in the sense that SIRT1 can deacetylate LXRs and promote their ubiquitination, thereby regulating ABCA1 and SREBP1c, which are involved in cellular cholesterol homeostasis (30). Studies have discovered that metformin can alleviate hepatic inflammation by activating CH25H in a SIRT1-dependent manner in that CH25H can promote cholesterol catabolism and the subsequent increased levels of 25-HC can inhibit inflammation driven by IL-1β. Moreover, metformin can suppress the polarization of M1 macrophages in a SIRT1-dependent manner (32). These findings suggest that SIRT1 gets involved in the regulation of cholesterol homeostasis through the LXR/ABCA1 pathway relevant to the regulation of CH25H and its downstream products.

The helper T cell plays an important role in the pathogenesis of IBD. The activation of LXRs leads to an increase in the expression of SREBP1, which then translocate into the nucleus and disrupts aryl hydrocarbon receptor (AHR), an essential positive regulator of Th17 cell differentiation. As a result, this negatively regulates the Th17 cell differentiation and inhibits the IL-17 promoter to reduce the IL-17 transcription (20, 33) (**Figure 1C**). 25-HC can also inhibit the differentiation of Th17 cells by suppressing the production of IL-1, which works synergistically with transforming the growth of factor-β (TF-β) to induce Th17 cells (28, 34). Jakobsson et al. (17) observed that LXR-deficient mice are more susceptible to suffer dextran sodium sulfate (DSS)- and 2,4,6-trinitrobenzene sulfonic acid (TNBS)-induced colitis and showed a slower recovery and decreased survival, while LXRs agonists can inhibit the expression of inflammatory cytokines, i.e. TNFα, and the recruitment of CD11b+ immune cell populations, and reduce the infiltration of pro-inflammatory Th17 cells in the colon epithelium. The activation of LXRs can also induce the differentiation of regulatory T cells, thereby reducing the severity of colitis (35). It has been found that the LXR level in the colon of IBD patients is significantly repressed, indicating the involvement of LXR in the development of IBD (17). 25-HC acts as a natural agonist of LXR, showing potential therapeutic effects for IBD.

### 3.2 GPR183

GPR183, also known as Epstein-Barr virus-induced G-protein-coupled receptor 2 (EBI2), is activated by its ligand 7α,25-dihydroxycholesterol (7α,25-HC). CH25H and Cytochrome P450 family 7 subfamily B member 1 (CYP7B1) are critical enzymes required for the generation of 7α,25-HC, which binds to GPR183 to exert its immune regulatory function by inducing the localization of immune cells in the spleen and lymph nodes. GPR183 is expressed in B cells, T cells, DCs, macrophages, natural killer (NK) cells, neutrophils, and plays an important role in immune system (10, 36).

**Figure 2:**
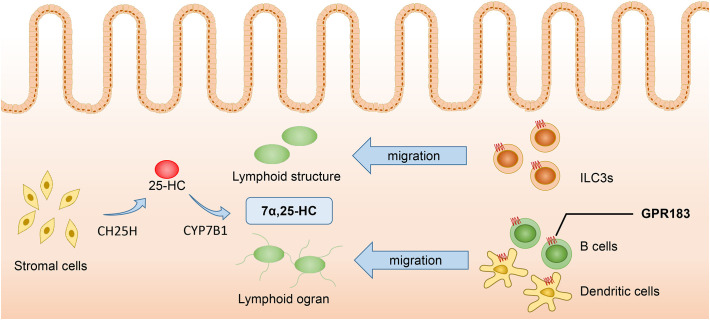
7α,25-HC inducing the migration of GPR183+ cells. Stromal cells serve as the primary source of 7α,25-HC, which is synthesized from cholesterol through hydroxylation by CH25H and CYP7B1 enzymes. Immune cells such as ILC3s, B cells, and DCs, migrate to colonic lymphoid tissues and lymphoid organs in response to the concentration gradient of 7α,25-HC. Proper localization of immune cells enhances the effectiveness of adaptive immune responses. 7α,25-HC, 7α,25-dihydroxycholesterol; GPR183, G protein-coupled receptor 183; CH25H, Cholesterol-25-hydroxylase; CYP7B1, Cytochrome P450 family 7 subfamily B member 1; ILC3s, Type 3 innate lymphoid cells.

The concentration of 7α,25-HC is a key factor inducing the migration of GPR183+ cells. Immune cells such as B cells, T cells, DCs, etc., migrate to lymphoid tissues and lymphoid organs according to the concentration gradient of 7α,25-HC, thus enhancing the efficacy of adaptive immune responses (37). Dietary cholesterol absorption and microbiome recognition can result in the production of 7α,25-HC in duodenal intestinal epithelial cells. The elevated levels of 7α,25-HC can influence the migration and positioning of intestinal plasma cells by binding to GPR183. Additionally, 7α,25-HC can reduce the expression of CD98 in these cells and impair their ability to secrete IgA (38). Innate lymphoid cells (ILCs) also express GPR183 and migrate to the solitary intestinal lymphoid tissue in colonic and small intestinal tissues according to the concentration gradient of 7α,25-HC. Among the different types of ILCs, ILC3s are dominant and express higher levels of GPR183. They are characterized by the expression of RORγt and can produce IL-22, playing an protective role in intestinal barrier integrity and immunity (37). Liu et al. (10) found that under the LPS stimulation, CH25H and CYP7B1 were highly up-regulated in spleen DCs, macrophages, and especially NK cells and neutrophils, indicating that these cells are likely to be the source of 7α,25-HC production. Additionally, since these cells also express GPR183, they may also be regulated by 7α,25-HC. Chen et al. (39) have recently discovered that lymphatic stromal cells are the primary source of 7α,25-HC within lymphoid organs, thus facilitating the migration of B cells and DCs, and stimulating the effective adaptive immunity and antibody production. Similarly, fibroblast stromal cells within the colonic lymphoid tissue can produce 7α,25-HC, which induces the migration of ILC3 and helps maintain colonic immune homeostasis (40) (**Figure 2**).

Despite its importance in regulating intestinal immune homeostasis, the overexpression of GPR183 is considered a pathological mechanism in colitis. Emgard et al. (40) found that GPR183 facilitates the formation of cryptopatches and isolated lymphoid follicles in the colon by ILC3s, both in steady-state conditions and during inflammation. The increase levels of GPR183 ligand 7α,25-HC produced by CH25H and CYP7B1 can exacerbate the inflammatory response through the GPR183-dependent activation of ILC migration, the recruitment of myeloid cells, and tissue remodeling, and knocking out of GPR183 was able to alleviate the severity of CD40-Ab-induced colitis. Wyss et al. (41) further investigated the function of GPR183 and found that GPR183 also plays a role in promoting lymphoid tissue formation in IL-10-/- colitis. However, in the DSS colitis model, GPR183 knockout resulted in a reduction of colonic lymphoid structures but did not alleviate the severity of acute or chronic inflammation induced by DSS. Therefore, it is speculated that the destruction of the epithelial barrier and innate immune response are the main factors involved in the pathogenesis of the DSS colitis model, while GPR183 plays a minor role. On the other hand, in ILCs-related colitis models such as CD40 colitis, IL-10 colitis, T-bet (-/-) Rag2 (-/-) ulcerative colitis (TRUC), GPR183 is one of the pathogenic mechanisms.

The 7α,25-HC-GPR183 pathway is critical for the formation of colonic lymphoid structures, but it is dispensable in small intestine, as other factors can compensate for its absence (40). Although GPR183 primarily influences ILCs-related colitis models, it is considered a potential therapeutic target for the treatment of IBD.

### 3.3 ROR

RORs belong to the nuclear receptor family of transcription factors binding oxysterols, which includes three different isoforms: RORα, RORβ, and RORγ. RORγ also has variants, including RORγ1 and RORγ2 (also known as RORγt) (42). RORγt participates in the differentiation process of IL-17-producing cells such as Th17 cells and γδT cells, and plays an important role in various pathological conditions. Oxysterols such as 20α-HC, 22R-HC, 25-HC, and 27-HC can act as the agonists of RORγ nuclear receptors, especially the RORγt isoforms, thereby affecting the function of lymphocytes (43). Soroosh et al. (44) found that 7β,27-HC and 7α,27-HC can promote the differentiation of Th17 cells through RORγt, confirming that oxysterols can directly regulate lymphocyte differentiation through RORγt. Therefore, 25-HC, a ROR receptor agonist, also appears to be involved in the differentiation of IL17-producing lymphocytes (20, 45). In contrast, Cui et al. (33) found that the activation of LXR can lead to a decrease in the level of RORγt expressions, inhibiting the differentiation of Th17 cells (**Figure 1C**). Indicating the dual role of 25-HC in immune system.

The generation and function of ILC3s depend on the expression of RORγt and are involved in the pathogenesis of IBD. The activated DCs produce IL-6, IL-23 and TNF-α, which promote the movement of ILC3s into or out of cryptopatches, starting an inflammatory immune cascade and causing intestinal inflammation (46, 47). 25-HC can inhibit the production of pro-inflammatory cytokines in macrophages and DCs, as well as the expression of RORγt in ILC3, thereby potentially impacting the occurrence and development of IBD.

### 3.4 Lipid metabolism and immune cell function regulated by 25-HC through SREBP

**Figure 3:**
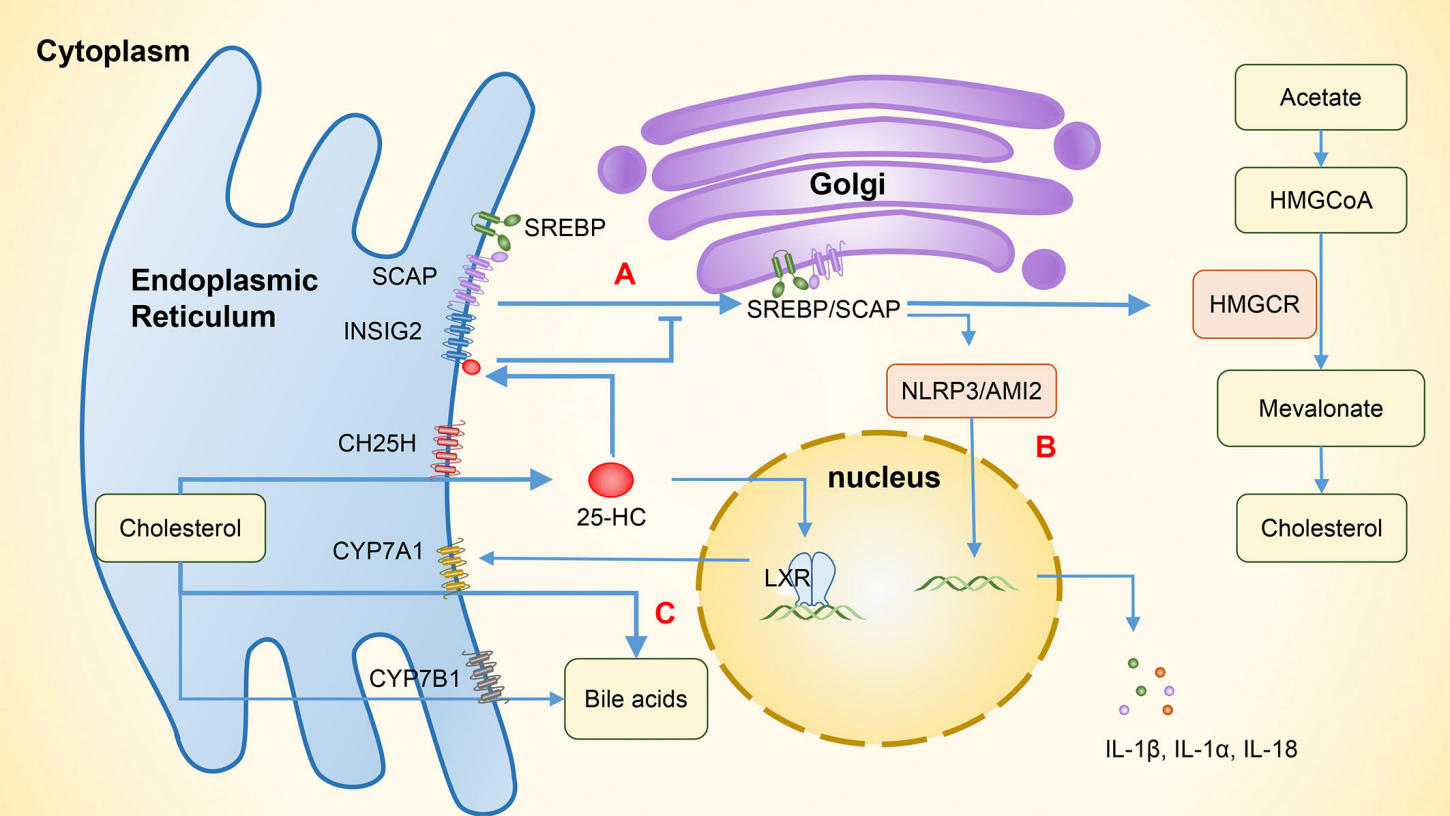
The involvement of 25-HC in the regulation of lipid metabolism and related immunity. **(A)** The binding of 25-HC with INSIG2 protein forms the SREBP/INSIG2/SCAP complex, which inhibits the transportation of SREBP to the Golgi apparatus. As a result, the expression of HMG-CoA reductase is suppressed and intracellular cholesterol production is reduced. **(B)** By inhibiting SREBP, the cholesterol synthesis is reduced, thereby inhibiting the activation of NLRP3 and AIM2 inflammasomes and subsequently suppressing the expression of IL-1β, IL-1α, and IL-18. **(C)** 25-HC increases the expression of CYP7A1 by activating LXR and enhances the synthesis of bile acids through the classical pathway. In addition to its role in hydroxylating 25-HC to 7α,25-HC, CYP7B1 also functions as an enzyme involved in the alternative bile acid pathway. 25-HC, 25-hydroxycholesterol; 7α,25-HC, 7α,25-dihydroxycholesterol; INSIG2, Insulin-induced gene 2, SREBP, Sterol regulatory-element binding protein; SCAP, SREBP cleavage-activating protein; HMG-CoA, 3-hydroxy-3-methyl glutaryl coenzyme A reductase; HMGCR, HMG-CoA reductase; NLRP3, NOD-like receptor protein 3; AIM2, Absent in melanoma 2; LXR, Liver X receptor; CYP7A1, Cytochrome P450 family 7 subfamily A member 1; CYP7B1, Cytochrome P450 family 7 subfamily B member 1.

SREBPs regulate genes that are involved in lipogenic processes, such as fatty acid and cholesterol synthesis. There are three isoforms of SREBPs, of which SREBP1a and SREBP1c are mainly involved in genes that relate to fatty acid synthesis, while SREBP2 regulates cholesterol synthesis (20). The overexpression of CH25H can suppress cholesterol biosynthesis through SREBP. In the absence of cholesterol, SREBPs bind to the SREBP cleavage-activating protein (SCAP) in the endoplasmic reticulum to form SREBP/SCAP complexes. These complexes are then transported to the Golgi apparatus and cleaved into mature transcription factor forms by site 1 protease (S1P) and site 2 protease (S2P), which promotes the expression of the rate-limiting enzyme, HMG-CoA reductase, and increases cholesterol biosynthesis. In the presence of excessive cholesterol, 25-HC binds to insulin-induced gene 2 (INSIG2), which is an endoplasmic reticulum anchor protein. This binding promotes the formation of SREBP/INSIG2/SCAP complexes that prevent SREBP from transporting to the Golgi apparatus, reducing intracellular cholesterol production (**Figure 3A**) (3, 20).

25-HC can inhibit the activation of NOD-like receptor protein 3 (NLRP3) inflammasomes and the expression of cytokines IL-1β, IL-1α, and IL-18 by suppressing SREBP translocation (28). Dang et al. (48) have shown that the cholesterol overload in macrophages can trigger the release of mitochondrial DNA and activate the absent in melanoma 2 (AIM2) inflammasomes, leading to an increase in IL-1β production. However, 25-HC can maintain the integrity of mitochondria by inhibiting SREBP2-mediated cholesterol synthesis, which in turn prevents the activation of AIM2 inflammasomes and the IL-1β-induced inflammation (**Figure 3B**). LPS activates macrophage TLR4 to induce the synthesis and secretion of 25-HC. This can inhibit the activity of the transcription factor SREBP2 in intestinal germinal center B cells, suppressing their differentiation into IgA plasma cells and reducing the production of IgA, and consequently impairing antigen-specific IgA responses during intestinal infections (12, 49). SREBPs also play a significant role in Th17 cell differentiation. Cholesterol synthesis induced by SREBPs increases intracellular sterol that serves as RORγ receptor agonists. By inhibiting SREBPs and reducing cholesterol metabolism, 25-HC can indirectly inhibit Th17 cell differentiation (45, 50). In addition, 25-HC can activate SREBP1 through LXRs, which can bind to the IL-17 promoter and interact with the positive regulator of Th17 differentiation, leading to the inhibition of Th17 cell differentiation and transcriptional antagonism of IL-17 (20, 33). As 25-HC has a weak but specific RORγ receptor activity, it can influence Th17 cell differentiation through various pathways (45).

When ER cholesterol is more than 5 moles% of total ER lipids, INSIG binds to SCAP/SREBP complex, preventing the complex from transporting to the Golgi and reducing the cholesterol synthesis and uptake. However, ER only contains about 1% of the total cellular cholesterol and no more than 5% of total membrane lipids. Similarly, 25-HC accounts for a small portion of the total cellular sterol; it can still regulate cellular lipid metabolism quickly and accurately (51). Das et al. (51) proposed that cholesterol in plasma membrane can be divided into three pools: accessible pool, sphingomyelin (SM)-sequestered pool, and essential pool. Of these, the accessible pool is labile, serving as a primary site for the excessive cholesterol absorption. Subsequently, this excess cholesterol is transported to the pool in endoplasmic reticulum, thereby regulating cholesterol metabolism. Abrams et al. (52) found that 25-HC produced by IFN-γ-stimulated macrophages can increase the activity of acyl-CoA: cholesterol acyltransferase (ACAT), which can convert cholesterol in the ER to cholesteryl esters and induce the formation of cholesterol-rich lipid droplets. This decreases the level of the free cholesterol in the ER, triggering the internalization of accessible cholesterol from the plasma membrane and reducing the cholesterol synthesis and uptake. In addition, 25-HC can result in a long-term suppression of accessible cholesterol through inhibiting SREBP2. The cell surface accessible cholesterol is crucial for bacteria to penetrate adjacent cells, and the antibacterial activity of 25-HC is based on the decrease in accessible cholesterol on the plasma membrane (52). Ormsby et al. (53) conducted further research and found that by reducing accessible cholesterol on the plasma membrane through ACAT, 25-HC can limit pore formation and cytolysis caused by pore-forming toxins, thus protecting tissues from pathogenic bacteria.

CH25H exerts its antivirus function through inhibiting cholesterol biosynthesis, interacting with viral components, and modulating inflammation and immunity (3). Gastrointestinal symptoms are also observed in patients with SARS-CoV-2 when the virus infects intestinal epithelial cells. This is because differentiated enterocytes express high levels of the SARS-CoV-2 receptor, angiotensin-converting enzyme 2 (ACE2) (54). Studies have shown that 25-HC engages in anti-SARS-CoV-2 activities. Wang et al. (55) proposed that 25-HC inhibits the fusion of the viral envelope with the plasma membrane by activating ACAT, which can reduce the accessible cholesterol in the plasma membrane. In a similar vein, the research conducted by Zang et al. (56) demonstrated that 25-HC is capable of reducing the fusion of viral membrane by inhibiting Niemann-Pick C1 (NPC1), which is responsible for the reduction of accessible cholesterol in the membrane of the endosomal/lysosomal compartment. However, SARS-CoV-2 infection can also increase the level of 7α,25-HC in lungs, attracting monocytes/macrophages through a GPR183-dependent mechanism and subsequently leading to inflammation. Inhibiting GPR183 can reduce SARS-CoV-2 loads, macrophage infiltration, and inflammatory cytokine expression (57).

Bile acids are synthesized in the liver from cholesterol. Evidence shows that the altered composition of primary bile acids and secondary bile acids may contribute to the development of IBD through differential effects on epithelial and immune cells (58). Metabolomics studies have found that primary bile acids (PBAs) are increased while secondary bile acids (SBAs) are decreased in patients with IBD (58, 59). Dong et al. (60) have shown that 25-HC can increase bile acid synthesis through the activation of LXRs, which is regulated by the classic pathway of CYP7A1, and secondary bile acids also increase due to the involvement of gut bacteria. In addition, CH25H can regulate innate immune responses via the SREBP regulation and alleviate the liver inflammation caused by a high-fat diet, indicating that CH25H has a regulatory role in bile acid metabolism and anti-inflammation (**Figure 3C**). In addition to hydroxylating 25-HC to 7α,25-HC, CYP7B1 is also an enzyme involved in the alternative bile acid pathway (61). In humans, the major SBAs are lithocholic acid (LCA) and deoxycholic acid (DCA). Studies (62, 63) have found that gut bacteria and corresponding enzymes can convert the LCA into 3-oxoLCA and isoLCA, which have different effects on the differentiation of immune cells. Specifically, 3-oxoLCA can inhibit the differentiation of Th17 cells via RORγt, while isoLCA promotes the differentiation of Treg cells by inducing the production of mitochondrial reactive oxygen species (mitoROS) and subsequently increasing the FoxP3 expression. These effects help reduce the inflammation in intestine, and both bile acid metabolites were found to be significantly reduced in patients with IBD. However, previous studies have shown that although CH25H can regulate the cholesterol metabolism in a tissue-specific fashion, it contributes little to the overall bile acid synthesis (64). Further research is needed to examine whether CH25H can help regulate the inflammatory responses in IBD patients by affecting the bile acid pathway.

### 3.5 Other mechanisms of immune regulation by CH25H

The research above indicates that 25-HC can inhibit the production of inflammatory factors through LXR or SREBP. In addition, 25-HC can also interact with myeloid differentiation protein 2 (MD2) to prevent LPS from binding to TLR4, thereby inhibiting the transcription of NF-κB and AP-1 and leading to the inhibition of inflammatory responses (65).

Regarding the pro-inflammatory effect of 25-HC, researchers (66, 67) have found that 25-HC can induce the secretion of pro-inflammatory cytokines and chemokines in macrophage cells, such as IL-1β and IL-6. When cocultured with smooth muscle cells and monocytic cells, 25-HC can induce monocytes to produce IL-1, which in turn synergistically increases the levels of IL-6 and MCP-1 expressions produced by smooth muscle cells (67, 68). Bai et al. (68) discovered that 25-HC can also enhance the IL-8 promoter activity caused by IL-1β in Caco-2 cells. Further research (69) has shown that 25-HC can bind to the lipid raft domains of the plasma membrane and promote Ca2+ influx, leading to the activation of calcium-dependent kinase PYK2, and consequently activating the MAPK ERK1/2 pathway and AP-1 transcription, which promoted the transcription of IL-8. Another study concluded that 25-HC promotes NLRP3 inflammasome assembly and its activation through potassium efflux, mitoROS and LXR-mediated pathways (70). Moreover, 25-HC can activate the FAK signaling pathway by binding to α5β1/αvβ3 integrins directly, leading to an increase in the production of pro-inflammatory cytokines, such as TNF and IL-6 (71).

Scholars (72, 73) have found that 25-HC can enhance the immune response mediated by TLR3 in airway epithelial cells, stimulate the release of IL-8 and IL-6 via the NF-κB pathway, and participate in neutrophilic airway inflammation. Friedrich et al. (74) have shown that for patients with IBD who did not respond to anti-TNF therapy or corticosteroids, their inflamed tissues were characterized by neutrophil infiltrates, fibroblast activation, and loss of epithelial cells. In addition, the high amount of IL-1β was expressed in colon ulcers, but not in NR3C1, ITGA4, or TNF. Fibroblast IL-1R signaling drives the recruitment of neutrophils, which then induces inflammation, indicating that targeting the neutrophil-attractant program in fibroblasts by blocking IL-1R could be used as an alternative treatment for patients with refractory IBD. CH25H can inhibit the secretion of IL-1β by macrophages and may have a potential role in regulating the migration and function of neutrophils, which are involved in the pathogenesis of IBD. However, there is a lack of relevant research on the mechanism by which CH25H regulates neutrophils.

CH25H can enhance the progression and metastasis of colorectal cancer (CRC). A reduced level of CH25H was observed in the cancer stroma, particularly in the intratumoral endothelial cells (ECs), among CRC patients (75). It is worth noting that intercellular biomolecule transfer (ICBT), facilitated by tumor-derived extracellular vesicles (TEVs), is also a crucial factor that influences the progression and prognosis of CRC. CH25H is capable of inhibiting of the fusion lipid membrane, thereby hindering the uptake of TEVs. As a result, CH25H restricts the ICBT-induced angiopoietin-2 (ANGPT2)-dependent activation of ECs, and inhibits intratumoral angiogenesis. Furthermore, it has also been observed that the administration of reserpine as a treatment can enhance the expression of CH25H in TEV-treated cells. This subsequently leads to a reduction in the ICBT between malignant and benign cells, ultimately resulting in the suppression of angiogenesis (75). Additionally, a study has revealed that LINC01915, a type of long non-coding RNA, inhibited the uptake of TEVs by normal fibroblasts (NFs), cancer-associated fibroblasts (CAFs) activation, and tumor angiogenesis through the miR-92a-3p/KLF4/CH25H axis, thereby impeding tumor growth. These findings indicate that CH25H has an inhibitory effect on CRC (76).

It is accepted that 25-HC as a soluble factor can function both as an autocrine and paracrine agent. Specifically, Doms et al. (77) employed transient transfection to conduct an exogenous expression of CH25H in HeLa cells using a plasmid. They observed that the exogenous expression of CH25H reduced the proportion of cells infected with reovirus, particularly in the cells expressing CH25H. Moreover, they found that reovirus infection decreased in cells that do not express CH25H, indicating that 25-HC may be secreted from cells to limit infection in CH25H non-expressing cells. On the other hand, Canfrán-Duque et al. (78) have found that in atherosclerosis, activated macrophages release 25-HC which acts on SMC through paracrine action. This interaction blunts SMC migration by altering platelet-derived growth factor (PDGF) signaling and ultimately promotes plaque instability. Thus, the potential role of extracellular 25-HC in intestinal immunity needs to be further explored.

CH25H may have either pro-inflammatory or anti-inflammatory effects, depending on the experimental protocols used, such as the concentration of 25-HC or treatment time. For instance, the high concentrations of 25-HC were used in the study that discovered its pro-inflammatory effect, while relatively low concentrations were used in the study that found its anti-inflammatory effect (28, 70).

## 4 The impact of CH25H on the occurrence and development of IBD

Although the pathogenesis of IBD is still unclear, it is mainly associated with genetic susceptibility, intestinal microbiota, environmental factors, and immunological dysregulation characterized by abnormal infiltration of T cells, B cells, macrophages, DCs, and neutrophils, which produce high levels of proinflammatory cytokines such as TNF, IL-1β, IFN-γ, and cytokines of the IL-23/Th17 pathway (15). As an oxysterol produced through oxidation by CH25H, 25-HC has various effects on immune cell regulation, but its specific role in IBD has not been fully ascertained.

### 4.1 The role of immune cells in IBD

#### 4.1.1 Macrophages

Macrophages are among the most abundant types of leukocytes, found in the intestines of all mammalian species. Macrophages play a crucial role in maintaining local homeostasis and the balance of commensal microbiota. Moreover, they are significant factors in the development of IBD (79, 80). Resident macrophages in the lamina propria of the intestine have the ability to capture and degrade bacteria in a non-inflammatory manner. This helps prevent commensal bacteria from crossing the intestinal epithelial barrier. Additionally, macrophages can maintain intestinal homeostasis through the production of anti-inflammatory cytokines like IL-10 and TGF-β, debris scavenging, angiogenesis, and wound repair (79–81). Intestinal macrophages are also capable of inducing T cells to become anergic or differentiate into Tregs to promote immune tolerance, and mediate Th1, Th2, and Th17 to help participate in the adaptive immune response (80).

In colitis models, monocytes and immature macrophages can migrate to intestinal mucosa through the CCL2 or MCP-1 mediated recruitment and produce large quantities of inflammatory mediators such as IL-1, IL-6, and TNFα, as well as inflammatory chemokines like CCL2 and CCL3 to coordinate the recruitment of other innate and adaptive immune cells, such as neutrophils, Th1, and Th17 cells (82). In IL-10-/- mice with spontaneous chronic colitis, macrophages differentiate into pro-inflammatory subsets that produce large amounts of IL-12 and IL-23 in response to bacterial stimulation, inducing Th1 cell polarization (81). Similar findings have been observed in patients with IBD. The inflamed mucosa of patients with IBD shows an increase in the number of macrophages, and several phenotypic and functional characteristics of these macrophages differ from those under physiological conditions. For example, these macrophages display the expression of T cell costimulatory molecules like CD40, CD80, and CD86 (83), as well as pathogen-associated molecular pattern (PAMP) receptors like TLR2, TLR4, CD89, TREM1, and CD14 (84, 85). The abnormal expression of CD14 in macrophages enhances their pro-inflammatory activities induced by LPS, ultimately leading to the secretion of the significant amounts of IL-23 and TNF-α, which further promotes the release of IFN-γ by lamina propria mononuclear cells. IFN-γ then drives macrophage differentiation toward an IL-23-hyperproducing phenotype and forms a positive feedback, thereby playing a pivotal role in the pathogenesis of IBD (84, 86).

#### 4.1.2 ILCs

ILCs are an important component of intestinal organs and contribute to antibacterial defense, immune regulation, maintenance of barrier function, and intestinal homeostasis (87). There are three subtypes of ILCs: type 1 ILCs (including NK cells and ILC1), type 2 ILCs (ILC2), and type 3 ILCs (including ILC3 and lymphoid tissue-inducing cells (LTis)) (47). Unlike intestinal B, T, and NK cells, the intestinal population of ILCs does not continuously replenish from circulation (88). They are maintained by self-renewal in physiological conditions and continuously produce cytokines and other soluble factors that can have a direct impact on the function of epithelial cells at steady state, such as ILC1 producing IFNγ, ILC2 producing IL-5 and IL-13, and ILC3 producing IL-22 and IL-17 (47, 87). Type 1 ILCs are predominantly located in upper gastrointestinal tract, type 2 ILCs are distributed over the intestine in relatively small proportions, and type 3 ILCs are mainly found in ileum and colon (89). Any imbalance in ILC subtypes can result in the disruption of intestinal homeostasis and lead to intestinal inflammation.

In patients with CD, the number of IL-17/IL-22-producing type 3 ILCs is reduced in inflamed intestinal tissues, while there is an accumulation of IFNγ-producing type 1 ILCs. This could be ascribed to a decrease in the expression of RORγt which is a marker for type 3 ILCs, followed by the expression of T-bet, NK1.1, and NKp46, resulting in the acquisition of type 1 ILC phenotype (46). Type 1 ILCs produce a significant amount of IFN-γ, which induces the migration of neutrophils and activates lymphocytes, macrophages, endothelial cells, and affects the tight junction function, resulting in the damage to the epithelial barrier, thereby exacerbating the induction and progression of inflammation (46). RORγt type 3 ILCs also involve in the pathogenesis of IBD. When stimulated by TNF-α, IL-23, and IL-6, RORγt type 3 ILCs enter and exit crypts, which may initiate inflammatory immune cascades that lead to intestinal inflammation (46, 47). The increase of IFN-γ–producing ILC1s and IL-17–producing ILC3s, and the decrease of IL-22–producing ILC3s, are associated with the level of inflammation in patients with IBD.

#### 4.1.3 T cells and DCs

CD4+ Th cells play a crucial role in adaptive immune response. Naive CD4+ T cells differentiate into various types of Th cells in response to different cytokines. Under the stimulation of IL-12 or IL-27, they differentiate into Th1 cells which primarily secrete IFN-γ. In contrast, under the stimulation of IL-4, they differentiate into Th2 cells which produce a range of interleukins, including IL-4, IL-5, IL-13, and IL-25. When exposed to both IL-4 and TGF-β, they differentiate into Th9 cells, which secrete IL-9, IL-10, and IL-21. Conversely, with the stimulation from IL-1, IL-6, IL-23, and TGF-β, they differentiate into Th17 cells that secrete IL-17A, IL-17F, IL-21, and IL-22. Finally, with the induction of IL-2 and TGF-β, they develop into Treg cells that maintain immune tolerance and regulate the homeostasis, activation, and function of lymphocytes (90, 91). Studies have shown that Th1 cells are primarily involved in the development of CD, while Th2 cells are associated with UC (92, 93). Additionally, the pathogenesis of UC involves the participation of Th9 cells (94), and both Th17 and Treg cells contribute to the pathogenesis of UC and CD (95). In IBD, the chronic inflammatory environment and the local hypoxic condition result in the upregulation of hypoxia-inducible-factor-1-alpha (HIF-1α). This upregulation can impair Th17 regulatory responses to AHR ligation by increasing ABC transporter levels, thus promoting the pro-inflammatory phenotype in T cells (96).

DCs are regarded as the most potent professional antigen-presenting cells within human body. They possess the ability to internalize, process, and present antigens to T cells while also orchestrating innate and adaptive immune responses. The increased expression of chemokines and adhesion molecules observed in the intestinal mucosa of patients with IBD results in an accumulation of DCs in inflammatory sites (97). In patients with CD, the failure to control IL-12 secretion by the activated DCs will lead to undesirable Th1 inflammatory responses (98). In mouse colitis, DCs within the colonic lamina propria express higher levels of costimulatory molecules (CD40, CD80, and CD86) and generate increased amounts of IL-12p40 and IL-23p19, which ultimately combine to form IL-23, thereby promoting Th17 differentiation (99). Additionally, the intestinal tissues of patients with CD exhibit a decreased abundance of CD11c DCs, resulting in an enhanced ability to generate Th1/Th2/Th17 responses (100).

#### 4.1.4 Neutrophils

Neutrophils are the first line of defense in the innate immunity of the intestinal mucosa. They are the most abundant immune cells and can be quickly recruited to sites of infection or inflammation (100). Kuhl et al. (101) discovered that blocking neutrophil adhesion and migration or neutrophil depletion could exacerbate TNBS/DNBS-induced colitis in mice. This indicates that neutrophils are an important factor in mediating wound healing in IBD. Further research (102, 103) has revealed that neutrophils can maintain mucosal barrier function and immune response in the gut by depleting local oxygen and stabilizing the transcription factor HIF, or by altering nucleotide signaling to promote mucosal inflammatory resolution and epithelial restitution.

Neutrophils participate in the development of IBD. Neutrophils cause the damage to the epithelial barrier and inflammation by producing high levels of ROS. They also release proteases, proinflammatory cytokines, and mediators such as IL-8, TNF-α, and leukotriene B4, which further damages the epithelial barrier and recruit monocytes and additional neutrophils to the inflamed tissue (100, 104). Neutrophil extracellular traps (NETs) are released by neutrophils during the infection and inflammation as a protective response to inhibit foreign pathogens. However, they can also cause damage to the intestinal barrier and activate proinflammatory functions of neutrophils through the phosphorylation of Akt, ERK1/2, and p38 (104, 105). The level of NETs increases in the inflamed intestinal mucosa, blood, and stool of IBD patients, especially during the active stage of the disease (104). NETs can activate macrophages to release cytokines such as IL-1β, TNF-α, IL-6, induce the activation of platelets and intestinal epithelial cells, promoting colitis and thrombosis. This ultimately causes the damage to intestinal epithelial and vascular endothelial cells (106). Studies have also shown that butyrate, a microbial metabolite in the intestines, can improve intestinal inflammation by inhibiting neutrophil migration and NETs formation, as well as reducing pro-inflammatory mediator production (107).

### 4.2 CH25H impacting on the disease phenotype of IBD

Macrophages are the main source of 25-HC (9, 11). Through interactions with membrane receptors, nuclear receptors, and the regulation of lipid metabolism and other pathways, 25-HC can influence the differentiation and function of immune cells, including macrophages, T cells, B cells, DCs, and neutrophils. Additionally, 7α,25-HC can induce the migration of immune cells, such as ILCs, DCs, and B cells, as well as regulating gut immune homeostasis and inflammation. Therefore, CH25H is believed to be involved in the pathogenesis of IBD.

Except for being generated in cells and tissues through enzymatic or nonenzymatic reactions, oxysterols are also found in various foodstuffs, particularly in cholesterol-rich foods. The most commonly represented oxysterols in cholesterol-rich foods are 7-oxygenated sterols and 5,6-oxygenated sterols, while 25-HC is present in smaller amounts, and both dietary and endogenous oxysterols have potentially proapoptotic, pro-oxidant and cytotoxic effects, leading to the loss of epithelial colonic cells and the impairment of the intestinal barrier function (108–110).

Chalubinski et al. (111) noted that although 25-HC can cause slight damage to the integrity of Caco2 cell monolayers, it does not have a significant effect on cell viability or apoptosis. This suggests that 25-HC is not the primary factor disrupting epithelial barrier function. Guillemot et al. (112) discovered that in both the DSS or TNBS-induced mouse colitis model and patients with IBD, there is a disturbance in the enzymatic metabolism of oxysterols. CH25H levels increased in mouse colon tissue, plasma, liver, and human colon tissue, as did the 7α,25-HC. This indicates that the increase in CH25H may serve as a form of internal homeostatic regulation to alleviate inflammatory reaction. One of the main characteristics of IBD is the destruction of the intestinal barrier. The damaged intestinal barrier allows unrestricted entry of microbiota into both lamina propria and bloodstream (113). A study conducted by Sheng et al. (114) revealed that intestinal tight junction protein expression is reduced in CH25H-/- mice, which leads to the disruption of the intestinal epithelial barrier function and increases susceptibility to and severity of DSS-induced colitis, while supplementing exogenous 25-HC can alleviate colitis in mice and improve the integrity of the intestinal barrier. This suggests that CH25H may have a protective role in colitis and is associated with intestinal epithelial regeneration and tissue reconstruction. IBD is usually accompanied by dysbiosis, and persistent dysbiosis can worsen inflammation. Conversely, chronic inflammation contributes to dysbiosis by altering the oxidative and metabolic environment of the intestine (115). The administration of medications, such as anti-TNFα antibody therapy, for the treatment of IBD has been found to impact the composition of intestinal microbiota (116). While CH25H has been shown to improve the intestinal inflammation and the integrity of the intestinal barrier, its impact on the composition of intestinal microbiota remains unknown.

The SIRT1-CH25H pathway is one of the mechanisms that contribute to the metformin-induced alleviation of hepatic inflammation (32), and the nuclear receptor SIRT1 has been shown to play a role in the pathogenesis of IBD. Caruso et al. (117) demonstrated that SIRT1 is downregulated in the inflamed tissue of patients with IBD and colitis models by TNF-α and IL-21. T cells and macrophages deficient in SIRT1 are hyperactivated and produce significant amounts of inflammatory cytokines. Conversely, the activation of SIRT1 can reduce the acetylation of NF-κBp65 and subsequently decrease NF-kB activation, thereby decreasing the levels of inflammatory cytokines such as IFN-γ, IL-17A, and IL-21. However, SIRT1 also has deleterious effects on IBD. The Inhibition of SIRT1 may reduce the severity of colitis by promoting the production of Foxp3+T-regulatory cells, as well as paneth and goblet cells, which play crucial roles in maintaining gastrointestinal homeostasis (118). Therefore, SIRT1 imbalances in the immune system can result in the development and progression of IBD, and it is speculated that SIRT1 decreases the production of inflammatory cytokines by macrophage and Th1/Th17 cells through the activation of CH25H, and enhance the LXR/ABCA1 pathway to exert anti-inflammatory effects. Since SIRT1 serves as the upstream regulator of CH25H, the role of SIRT1 activators and inhibitors may influence the effects of CH25H on IBD relevant to gastrointestinal immune homeostasis. SIRT1 is relevant to the impact of CH25H on IBD through immune cells, the detection of both SIRT1 and CH25H can assist in the early diagnosis and treatment of the disease.

However, studies have shown that the overexpression of CH25H in ILC-related colitis models can stimulate the pro-inflammatory ILC3 activity and aggravate inflammatory reactions (40, 41). Additionally, 25-HC, the product of CH25H can contribute to the development of intestinal fibrosis, resulting in complications such as intestinal stenosis (119). In other conditions, such as obesity and diabetes, CH25H is upregulated in adipose tissue while associating with insulin resistance and adipose tissue inflammation. This is because 25-HC can induce inflammatory gene expressions in macrophages and preadipocytes from patients with diabetes (120). These results suggest that the role of CH25H may be different or even opposite in different disease models, possibly related to the pathogenesis and microenvironment of the disease.

## 5 Conclusions and perspectives

CH25H and its downstream products have been found to have various functions, such as immunomodulation and lipid metabolism. The downstream product of CH25H exerts its function by binding to multiple membrane receptors and nuclear receptors, such as LXR, GPR183 and ROR. This helps regulate the immune cell function in maintaining gastrointestinal homeostasis. Additionally, 25-HC can regulate the activity of SREBP, thereby inhibiting the biosynthesis of fatty acids and cholesterol and playing a role in lipid metabolism regulation. 25-HC can also regulate immune function via SREBP.

The immunological dysregulation caused by IBD is characterized by epithelial damage, the expansion of inflammation and the infiltration of various types of immune cells, including macrophages, ILCs, T cells, DCs and neutrophils. 25-HC can influence the differentiation and function of these immune cells, while 7α,25-HC can induce their migration. These effects of CH25H metabolite may be different or even opposite in different conditions. The dual role of CH25H may depend on the pathogenesis and microenvironment of IBD. This highlights the importance of maintaining CH25H balance in the immune system for the treatment of IBD.

The pathogenesis of IBD remains unclear, and the high expression of CH25H in patients with IBD makes it a potential target for future diagnosis and treatment. While the regulation of CH25H and its downstream products have been extensively investigated, its role and function in the immune system and related diseases are not fully understood. Although it has been confirmed that various immune cells play a role in the development of IBD, research on discussing the relationship between CH25H and IBD is still insufficient. Further investigation on the impact of CH25H on IBD through immune cells is essential for the early diagnosis and treatment of IBD.

## Author contributions

GZ wrote the first draft of the manuscript. CH, SW, CL contributed to writing-review and editing. ML supervised the study. All authors contributed to the article and approved the submitted version.
